# Delayed Closed Reduction and Percutaneous Fixation of a Neglected Volar Proximal Interphalangeal (PIP) Fracture-Dislocation Presenting 17 Days After Injury: A Two-Year Follow-Up

**DOI:** 10.7759/cureus.113055

**Published:** 2026-07-20

**Authors:** Ahmed Alsaedawy A Albeshlawy

**Affiliations:** 1 Orthopedic Surgery, Khafji General Hospital, Khafji, SAU

**Keywords:** closed reduction, delayed reduction, extension block pinning, fracture dislocation, hand trauma, neglected injury, percutaneous fixation, proximal interphalangeal joint, proximal interphalangeal joint dislocation, volar dislocation

## Abstract

Volar proximal interphalangeal (PIP) joint fracture-dislocations presenting more than two weeks after injury pose significant management challenges, as progressive soft tissue fibrosis and capsular contracture frequently necessitate open reduction. Reports of successful delayed closed reduction with long-term follow-up in this setting remain limited in the literature. We report an 18-year-old man who presented 16 days after injury with a neglected volar PIP fracture-dislocation of the left ring finger, managed with delayed closed reduction under sedation and fluoroscopic guidance the following day (17 days post-injury), followed by percutaneous fixation. At two-year follow-up, the patient achieved full PIP extension (0°), a flexion arc of 80°, and a QuickDASH score of 0/100, with no radiographic evidence of post-traumatic osteoarthritis. This case suggests that delayed closed reduction may remain feasible beyond two weeks in carefully selected patients, potentially avoiding open surgery while achieving satisfactory long-term functional and radiographic outcomes.

## Introduction

Fracture-dislocations of the proximal interphalangeal (PIP) joint are challenging injuries requiring restoration of both articular congruity and joint stability to preserve function [1]. Delayed or neglected injuries are associated with progressive fibrosis, contracture, chronic instability, and post-traumatic stiffness [2].

Volar PIP fracture-dislocations are less common than dorsal injuries. Unlike dorsal PIP fracture-dislocations, which usually involve the volar lip of the middle phalanx and are commonly related to volar plate disruption, volar fracture-dislocations involve the volar displacement of the middle phalanx. They are often associated with dorsal lip fractures. These injuries raise particular concern for central slip injury, extensor mechanism dysfunction, persistent instability, and later boutonnière-type deformity. For this reason, assessment of active extension and central slip integrity is important both before and after reduction.

Management of neglected cases remains controversial. Although open reduction is frequently advocated in delayed presentations, minimally invasive approaches may still be feasible in carefully selected patients [3]. Whether delayed closed reduction remains feasible beyond two weeks after injury has been poorly documented in the literature, and reports describing successful outcomes at two years following such management remain limited [4].

In this report, the term "neglected" is used to describe a presentation characterized by a prior failed reduction attempt and established soft tissue changes, rather than by a strict time threshold.

We report a neglected volar PIP fracture-dislocation presenting 17 days after injury, successfully managed with delayed closed reduction and percutaneous fixation, with satisfactory two-year clinical and radiographic outcomes. The key contribution of this case is that closed reduction succeeded despite a failed previous attempt, with durable outcomes confirmed at two-year follow-up.

## Case presentation

An 18-year-old man presented to the orthopedic clinic 16 days after sustaining a traumatic hand injury, with pain, swelling, deformity, and restricted motion of the left ring finger. The injury occurred during an assault; however, the precise mechanism of force could not be determined from the history provided by the patient. Prior to operative management, a colleague in the orthopedic clinic attempted a closed reduction under digital block, but it was unsuccessful.

**Figure 1:**
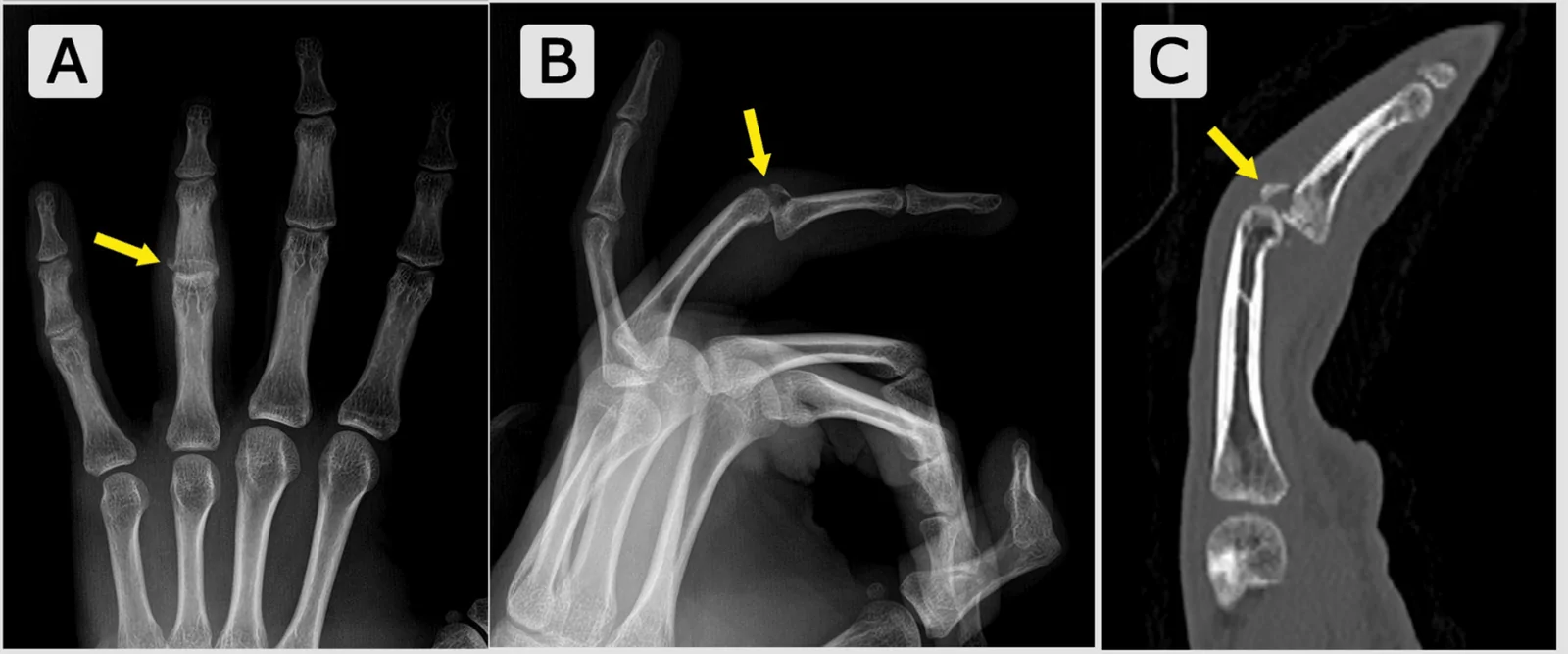
Preoperative imaging. (A) AP radiograph of the left hand demonstrating volar malalignment of the ring finger PIP joint (arrow). (B) Lateral radiograph confirming complete volar dislocation of the PIP joint (arrow). (C) Sagittal CT image demonstrating complete volar dislocation with a small dorsal rim fracture estimated to involve less than 30% of the articular surface (arrow) AP: anteroposterior, PIP: proximal interphalangeal, CT: computed tomography

On physical examination, marked swelling was noted around the PIP joint with dorsal tenderness on palpation. The finger was held in a fixed deformity of partial flexion, with painful, markedly restricted active and passive motion. Distal neurovascular examination was intact. Plain radiographs demonstrated volar dislocation of the PIP joint (Figure 1A-1B).

CT confirmed complete volar dislocation of the left ring finger PIP joint with a dorsal rim fracture involving the base of the middle phalanx, estimated to involve less than 30% of the articular surface, and no additional osseous injury (Figure 1C). On sagittal CT, apparent separation between the flexor tendon shadow and the phalangeal cortex was noted, raising the possibility of A3 pulley or flexor sheath involvement. This was not directly confirmed because the procedure was performed using a closed technique, with no open exploration.

Following evaluation, the patient and his guardian were counseled regarding the delayed presentation, guarded prognosis, risk of stiffness, and possible need for open reduction. Informed consent was obtained for operative management.

Surgery was performed the following day (17 days post-injury) under local anesthesia with sedation and fluoroscopic guidance. Closed reduction was achieved in a single controlled intraoperative maneuver with the PIP joint slightly flexed. Gentle longitudinal traction was applied to the ring finger, combined with direct dorsal pressure over the base of the middle phalanx to guide the joint back into anatomic alignment. Joint stability was confirmed clinically and fluoroscopically prior to K-wire insertion. Although the joint appeared concentrically reduced after manipulation, K-wire fixation was used because this was a delayed presentation with a failed prior reduction attempt and an associated fracture fragment, creating concern for recurrent instability during early motion. No repeated forceful manipulation was required. Open reduction instruments were kept available as a backup in case closed reduction proved unsuccessful. The successful reduction, despite the 17-day delay, may have been facilitated by the relatively small fracture fragment (estimated to involve less than 30% of the articular surface) and by soft tissue contracture insufficient to prevent closed reduction, as demonstrated by the intraoperative findings.

**Figure 2:**
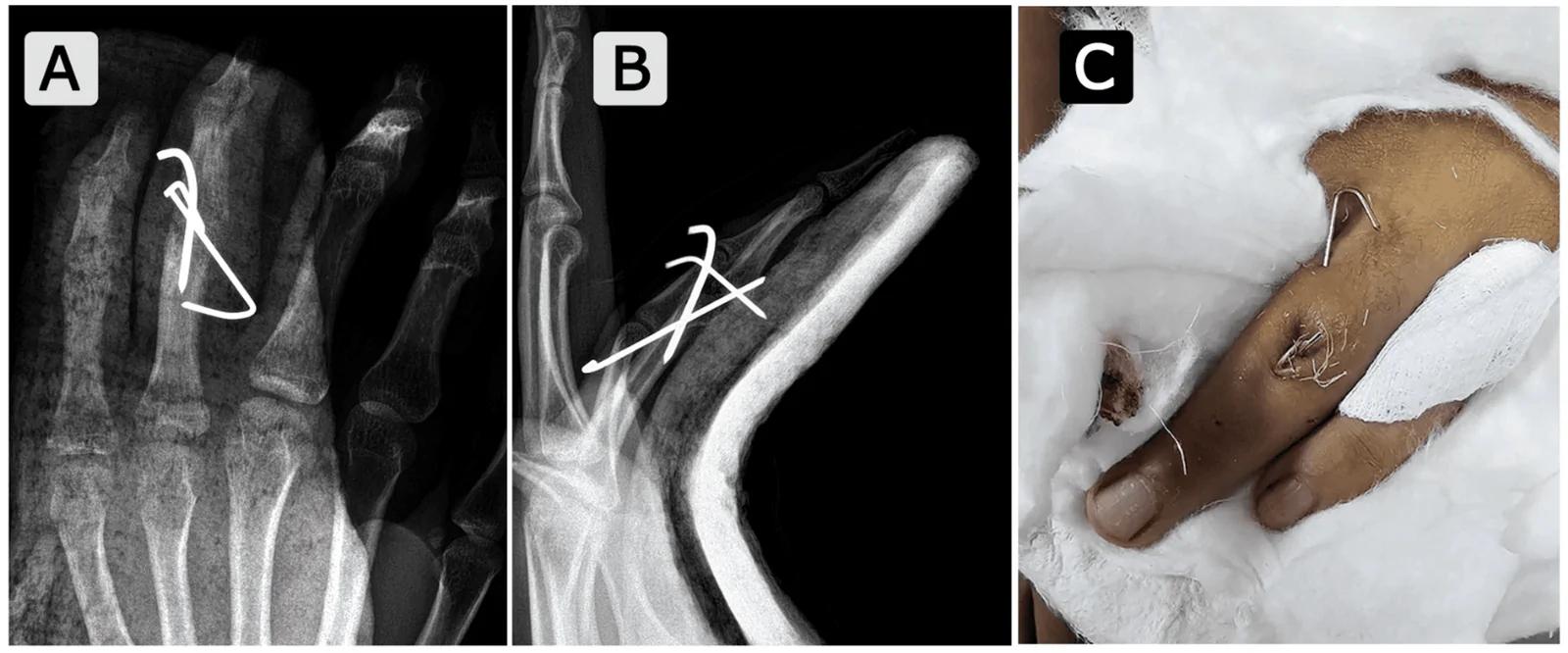
Early postoperative follow-up (13 days post-surgery). (A) AP and (B) lateral radiographs confirming maintained concentric reduction with the three K-wires in situ: extension block K-wire (1.5 mm), fragment fixation K-wire (1.2 mm), and transarticular stabilization K-wire (1.2 mm). (C) Clinical photograph demonstrating the K-wire configuration with the PIP joint maintained in full extension AP: anteroposterior

Percutaneous fixation consisted of three K-wires: an extension block K-wire (1.5 mm), a fragment fixation K-wire (1.2 mm), and a transarticular stabilization K-wire (1.2 mm). The decision to fix the fragment was not based solely on its size but on the intraoperative instability pattern observed, specifically, fragment mobility at the dorsal rim, a location critical to extensor mechanism function. Reduction and hardware placement were confirmed fluoroscopically, and a volar slab was applied with the PIP joint in full extension to maintain reduction (Figure 2A-2C).

At three weeks, the transarticular K-wire was removed, and early active mobilization was initiated. At five weeks, the remaining wires were removed following radiographic confirmation of union. A formal hand rehabilitation program was prescribed; however, the patient did not attend structured physiotherapy sessions.

At the two-year follow-up, the patient reported no pain, instability, or functional limitations. Mild cold-weather discomfort was noted but did not affect daily activities. Despite minor residual stiffness, the patient reported no functional limitations in any daily activities, including heavy gripping tasks. The QuickDASH score was 0/100, indicating no perceived disability.

**Figure 3:**
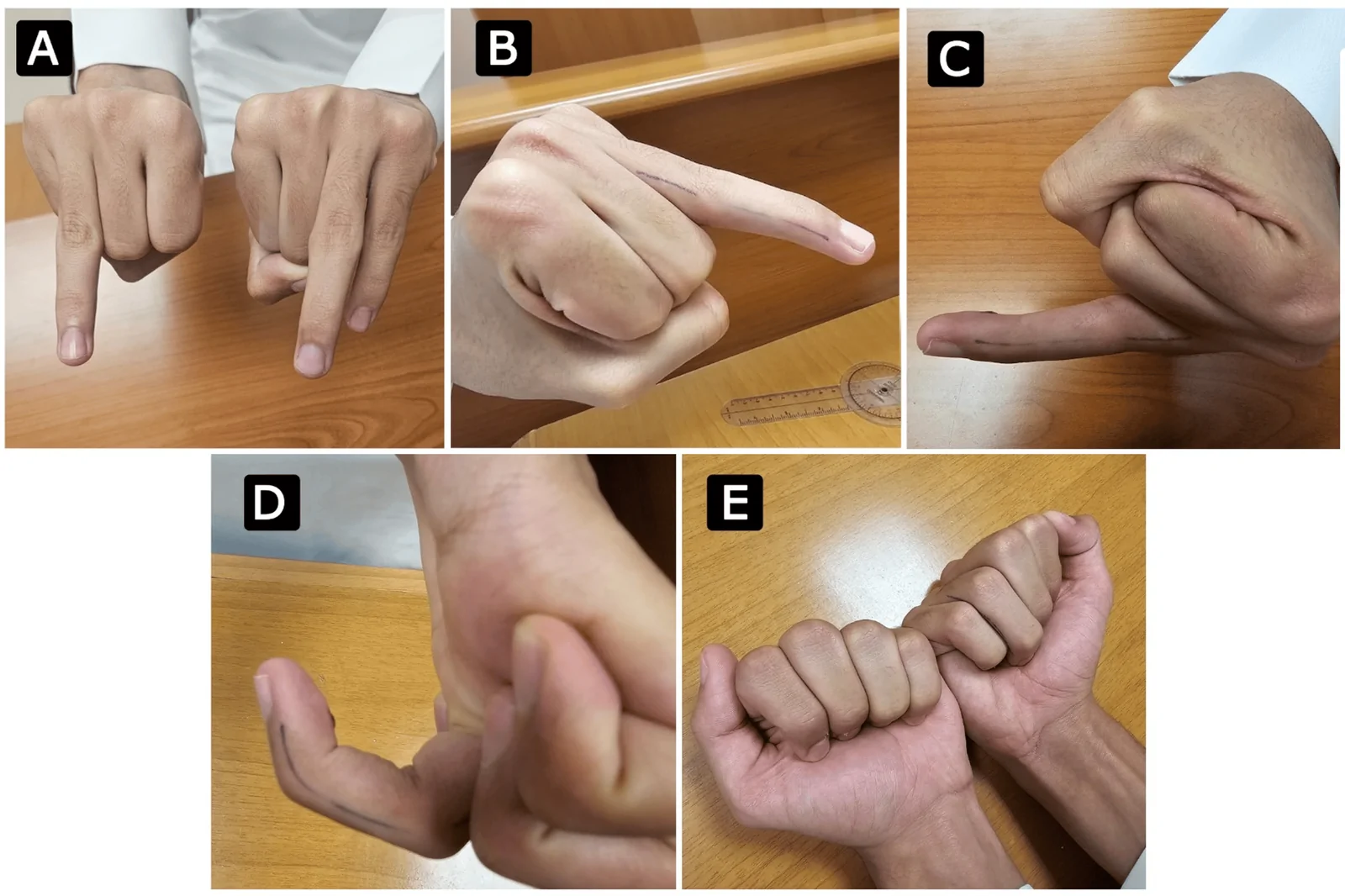
Two-year clinical follow-up. (A) Bilateral comparison demonstrating full active PIP extension (0°) without lag. (B) Dorsal view of the affected ring finger demonstrating full active extension. (C) Lateral view demonstrating full active PIP extension (0°) with no extension lag. (D) Lateral view demonstrating active PIP flexion arc (approximately 80°). (E) Bilateral grip comparison demonstrating satisfactory functional grip

Clinical examination demonstrated full active PIP extension (0°) with no extension lag, a PIP flexion arc of approximately 80° (0°-80°) measured by the treating surgeon using a standard handheld goniometer, and DIP flexion of approximately 50°. The contralateral ring finger was measured for comparison and demonstrated a PIP flexion arc of approximately 95° (0°-95°). The PIP joint was stable, and central slip integrity was confirmed on Elson testing. No tenderness or swelling was noted (Figure 3A-3E).

**Figure 4:**
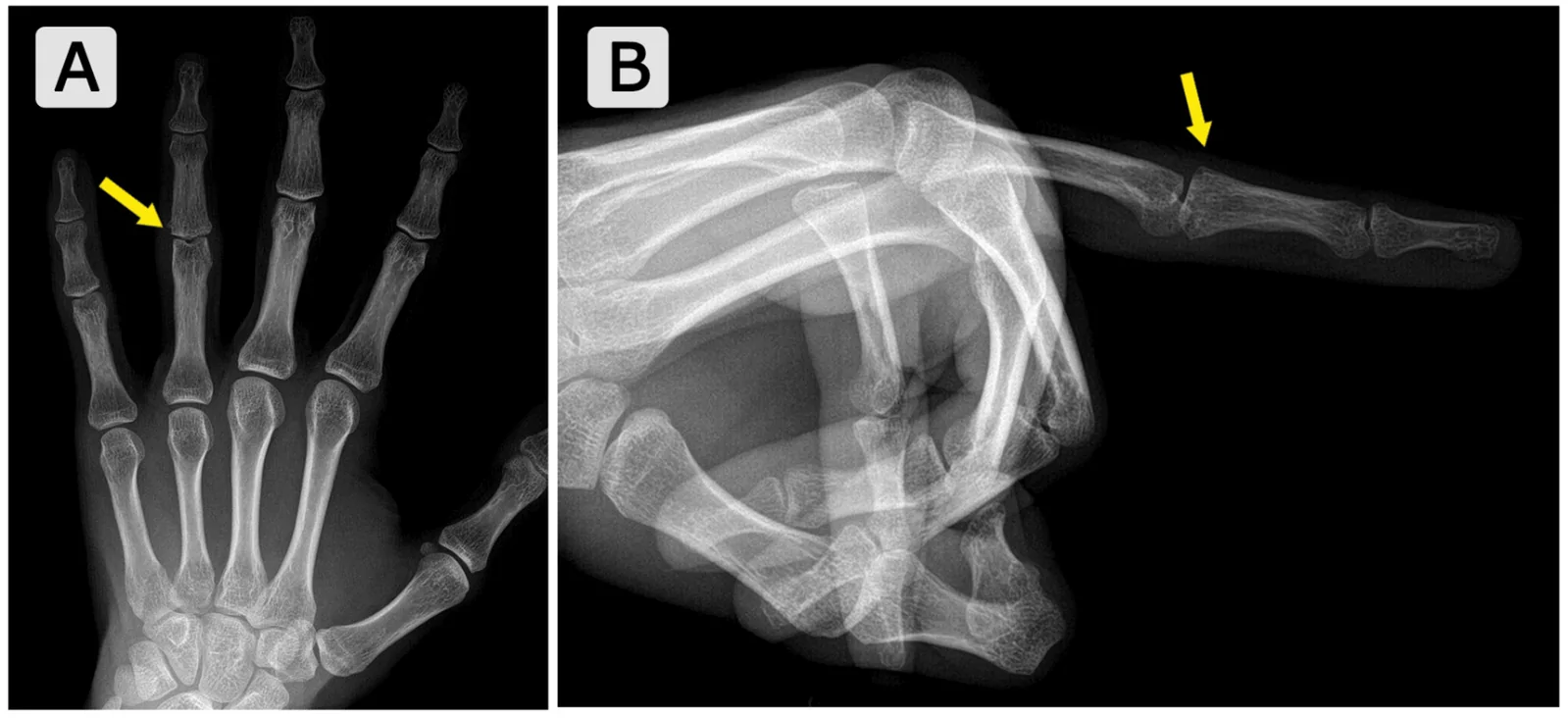
Two-year follow-up radiographs. (A) AP view demonstrating maintained reduction, preserved joint congruity, complete fracture union, and no radiographic evidence of post-traumatic osteoarthritis (arrow). (B) Lateral view confirming full active PIP extension (0°) with no extension lag and maintained joint alignment (arrow) AP: anteroposterior, PIP: proximal interphalangeal

Follow-up radiographs demonstrated maintained reduction, preserved joint congruity, complete fracture union, and no radiographic evidence of post-traumatic osteoarthritis or recurrent subluxation (Figure 4A-4B). Dynamic range of motion at two-year follow-up, demonstrating full active flexion and extension of the hand, is shown in Video 1.

**Video 1:** Two-year postoperative range of motion showing full active flexion and extension of the hand

## Discussion

Neglected PIP fracture-dislocations are challenging due to progressive fibrosis, contracture, and difficulty achieving closed reduction. Open reduction is frequently recommended in delayed cases; however, it carries risks of additional soft tissue trauma, devascularization, and postoperative stiffness [5].

The rarity of neglected volar PIP fracture-dislocations means that management recommendations are largely based on small series and expert opinion. Spinner and Choi first described the pathoanatomy of anterior PIP dislocation in 1970, highlighting the central slip rupture associated with these injuries [6]. Kiefhaber and Stern provided a comprehensive classification framework for PIP fracture-dislocations and emphasized the importance of restoring articular congruity [7]. Bamal and Bindra reported that open reduction of chronic PIP dislocations can yield acceptable outcomes but emphasized the technical difficulty and the risk of postoperative stiffness inherent to open dissection [8]. Lee and Teoh reported satisfactory outcomes with open reduction and interfragmentary screw fixation for delayed PIP fracture-dislocations, further highlighting the technical demands of open surgical management in these cases [9]. Houshian et al. demonstrated that single-stage distraction correction for neglected dorsal fracture-dislocations, at a mean of six weeks post-injury, achieved satisfactory results with an average range of motion of 79°, supporting the feasibility of minimally invasive management in delayed presentations [10].

In contrast, the present case demonstrates that delayed closed reduction under anesthesia with fluoroscopic guidance remained achievable at 17 days post-injury, consistent with the principle that a trial of closed reduction is warranted before committing to open surgery. Notably, successful reduction was achieved despite a previously unsuccessful outpatient reduction attempt under digital block, suggesting that delayed closed reduction under sedation and fluoroscopic guidance may remain feasible in selected patients even after a failed outpatient reduction attempt. The extension block pinning technique, originally described by Viegas [11], provided stable fixation while allowing early mobilization, both of which are recognized as essential for preserving long-term digital function [12]. Although central slip injury is commonly associated with volar PIP dislocations [6], clinical examination in this patient demonstrated preserved extensor mechanism function at final follow-up, with the Elson test remaining negative at two years. This observation suggests that central slip integrity may be preserved in some cases, particularly when the dislocation is associated with a small dorsal rim fracture rather than significant soft tissue disruption. To the best of our knowledge, reports describing successful delayed closed reduction of neglected volar PIP fracture-dislocations beyond two weeks after injury with long-term follow-up remain limited. This case contributes valuable long-term follow-up data to the limited literature on delayed closed reduction of neglected volar PIP fracture-dislocations. Table 1 summarizes key comparable studies in the literature.

**Table 1:** Summary of selected studies reporting outcomes of neglected or delayed PIP fracture-dislocations ROM: range of motion, PIP: proximal interphalangeal joint

Study	Delay	Treatment	ROM	Follow-up
Bamal and Bindra (2020) [8]	Chronic (>6 weeks)	Open reduction	Variable	Mean 28 months
Houshian et al. (2008) [10]	Mean 6 weeks	Distraction correction	Mean ROM 79°	Mean 14 months
Present case	17 days	Closed reduction + percutaneous K-wire fixation	PIP ROM 0°-80°	24 months; QuickDASH score 0

This case demonstrates that delayed closed reduction may remain feasible even after 17 days in selected patients. The successful reduction in this case may have been facilitated by several favorable factors: the small size of the dorsal rim fracture fragment (estimated to involve less than 30% of the articular surface), which minimized articular incongruity and soft tissue interposition; the relatively short duration of neglect (17 days), which likely limited the degree of periarticular fibrosis; and the use of continuous fluoroscopic guidance allowing precise manipulation. These factors collectively suggest that patient selection and fracture morphology are critical determinants of feasibility when attempting delayed closed reduction. Although the final PIP flexion arc (approximately 80°) was lower than that of the contralateral side (95°), the patient reported no functional limitation and achieved a QuickDASH score of 0/100, indicating that satisfactory functional recovery can be achieved even without full ROM restoration. Advantages observed included avoidance of open reduction, preservation of soft tissues, stable percutaneous fixation, and satisfactory long-term preservation of PIP motion. Notably, the satisfactory result was maintained at two years, with no radiographic evidence of post-traumatic osteoarthritis or instability, strengthening the rationale for attempting closed reduction under anesthesia before proceeding to open surgery in selected delayed presentations. The mild residual distal interphalangeal (DIP) joint stiffness observed at final follow-up is addressed in the Limitations section.

Limitations

MRI assessment of the central slip was not performed; therefore, central slip integrity was assessed clinically using the Elson test rather than advanced imaging.

This is a single case report, and no generalizable conclusions can be drawn [13]. Selection bias likely contributed to the satisfactory outcome, as the small fracture fragment, limited fibrosis, and the patient's age may collectively have facilitated successful reduction and healing. Similar attempts may not succeed in presentations with more advanced fibrosis or comminuted fractures. Goniometric measurements were performed clinically using a standard handheld goniometer; however, formal photographic documentation of the measurement position was not obtained, reflecting the practical challenges of managing patients with limited engagement with healthcare. Objective grip strength was not measured; however, the patient achieved a QuickDASH score of 0/100 and demonstrated satisfactory grip function on clinical examination. The absence of formal rehabilitation possibly contributed to mild residual DIP stiffness at final follow-up, underscoring the importance of structured hand therapy even after technically successful surgery. Future prospective studies are needed to define reliable selection criteria for minimally invasive management of neglected PIP injuries.

Clinical message

Surgical management of neglected volar PIP fracture-dislocation may still allow successful delayed closed reduction under anesthesia with fluoroscopic guidance beyond two weeks after injury in carefully selected patients, potentially avoiding the need for open surgery. Accordingly, a trial of closed reduction under anesthesia should be considered before proceeding to open reduction in appropriate cases of delayed presentation. Following percutaneous fixation, structured hand therapy is essential to optimize digital function, particularly DIP joint motion, even when PIP joint recovery is satisfactory [14]. In this case, the excellent functional outcome was reflected by a QuickDASH score of 0/100 at the two-year follow-up, indicating no perceived functional limitation [15].

## Conclusions

Neglected volar PIP fracture-dislocations may remain amenable to delayed closed reduction and percutaneous fixation beyond two weeks after injury. In appropriately selected patients, this minimally invasive strategy may avoid the need for open reduction while achieving satisfactory long-term functional and radiographic outcomes. A trial of delayed closed reduction under anesthesia may be considered prior to open surgery in carefully selected delayed presentations.
